# Cribriform and intraductal prostate cancer are associated with increased genomic instability and distinct genomic alterations

**DOI:** 10.1186/s12885-017-3976-z

**Published:** 2018-01-02

**Authors:** René Böttcher, Charlotte F. Kweldam, Julie Livingstone, Emilie Lalonde, Takafumi N. Yamaguchi, Vincent Huang, Fouad Yousif, Michael Fraser, Robert G. Bristow, Theodorus van der Kwast, Paul C. Boutros, Guido Jenster, Geert J. L. H. van Leenders

**Affiliations:** 1 Department of Urology, Erasmus MC, Rotterdam, the Netherlands; 2 Department of Pathology, Erasmus University Medical Center, Josephine Nefkens Institute building, Be-222, P.O. Box 2040, Rotterdam, 3000 CA The Netherlands; 3 Informatics & Biocomputing Program, Ontario Institute for Cancer Research, Toronto, ON Canada; 4 Department of Medical Biophysics, University of Toronto, Toronto, ON Canada; 5 Ontario Cancer Institute, Princess Margaret Cancer Centre, University Health Network, Toronto, ON Canada; 6 Department of Radiation Oncology, University of Toronto, Toronto, ON Canada; 7 Department of Pathology and Laboratory Medicine, Toronto General Hospital, University Health Network, Toronto, ON Canada; 8 Department of Pharmacology and Toxicology, University of Toronto, Toronto, ON Canada

**Keywords:** Cribriform, Intraductal carcinoma, Prostate cancer, Copy number alteration, Aggressive disease, Genomic instability

## Abstract

**Background:**

Invasive cribriform and intraductal carcinoma (CR/IDC) is associated with adverse outcome of prostate cancer patients. The aim of this study was to determine the molecular aberrations associated with CR/IDC in primary prostate cancer, focusing on genomic instability and somatic copy number alterations (CNA).

**Methods:**

Whole-slide images of The Cancer Genome Atlas Project (TCGA, *N* = 260) and the Canadian Prostate Cancer Genome Network (CPC-GENE, *N* = 199) radical prostatectomy datasets were reviewed for Gleason score (GS) and presence of CR/IDC. Genomic instability was assessed by calculating the percentage of genome altered (PGA). Somatic copy number alterations (CNA) were determined using Fisher-Boschloo tests and logistic regression. Primary analysis were performed on TCGA (*N* = 260) as discovery and CPC-GENE (*N* = 199) as validation set.

**Results:**

CR/IDC growth was present in 80/260 (31%) TCGA and 76/199 (38%) CPC-GENE cases. Patients with CR/IDC and ≥ GS 7 had significantly higher PGA than men without this pattern in both TCGA (2.2 fold; *p* = 0.0003) and CPC-GENE (1.7 fold; *p* = 0.004) cohorts. CR/IDC growth was associated with deletions of 8p, 16q, 10q23, 13q22, 17p13, 21q22, and amplification of 8q24. CNAs comprised a total of 1299 gene deletions and 369 amplifications in the TCGA dataset, of which 474 and 328 events were independently validated, respectively. Several of the affected genes were known to be associated with aggressive prostate cancer such as loss of *PTEN*, *CDH1*, *BCAR1* and gain of *MYC*. Point mutations in *TP53*, *SPOP* and *FOXA1*were also associated with CR/IDC, but occurred less frequently than CNAs.

**Conclusions:**

CR/IDC growth is associated with increased genomic instability clustering to genetic regions involved in aggressive prostate cancer. Therefore, CR/IDC is a pathologic substrate for progressive molecular tumour derangement.

**Electronic supplementary material:**

The online version of this article (10.1186/s12885-017-3976-z) contains supplementary material, which is available to authorized users.

## Background

Prostate cancer is heterogeneous regarding its pathologic features, genetic background and clinical outcome. Clinical-decision making mostly depends upon serum Prostate Specific Antigen (PSA) level, clinical tumour stage, and pathologic biopsy Gleason score (GS) – a grading system based on architectural tumour patterns [1]. While patients with the lowest GS ≤6 (WHO/ISUP group 1) have an excellent patient outcome, those with the highest GS 9–10 (WHO/ISUP group 5) have the worst [1, 2]. The clinical outcome of GS 3 + 4 = 7 (WHO/ISUP group 2) prostate cancer patients is variable. Improving risk assessment in this subgroup of patients is of clinical relevance as biopsy GS 3 + 4 = 7 is an important threshold for active treatment. Recent studies have indicated that, among Gleason grade 4 growth patterns, cribriform growth is associated with worse clinical outcome [3–6].

In recent years the clinical relevance of intraductal carcinoma of the prostate (IDC) – a malignant epithelial proliferation filling and extending pre-existent glands – has been acknowledged. Although not included in the Gleason grading system, IDC has been associated with high GS, advanced tumour stage, biochemical relapse and distant metastasis [7–12]. IDC often mimics invasive cribriform carcinoma, requiring basal cell immunohistochemistry for their distinction. Recently, our group has shown that patients with cribriform and/or intraductal carcinoma (CR/IDC), have significantly worse disease-specific survival probabilities than those without, regardless of GS [13]. Furthermore, patients with focal CR/IDC have similar outcome as men with extensive CR/IDC, indicating that the mere presence of this growth pattern is an adverse feature [13, 14].

Although the number of mutational events in prostate cancer is relatively low, copy number alterations (CNAs) are significantly more frequent [15–24]. Several studies have developed molecular prognostic signatures, showing that indolent tumours have relatively few CNAs in contrast to large-scale CNAs in high-grade or metastatic tumours [16, 17, 25, 26]. However, both the intra- and inter-tumour heterogeneity pose significant challenges for personalizing treatment in patients with prostate cancer [27–29]. For instance, GS 7 prostate cancers harbour a wide range of CNA burden varying between <1% to 50% [26].

Since presence of CR/IDC growth pattern is an independent, adverse clinico-pathologic parameter, we hypothesize that CR/IDC represents a morphological substrate of genomic alterations associated with aggressive disease [13]. The objective of this study was to determine the CNAs and single nucleotide variants (SNVs) associated with CR/IDC using bioinformatics analyses of datasets from The Cancer Genome Atlas Project (TCGA) and the Canadian Prostate Cancer Genome Network (CPC-GENE).

## Methods

### Pathological review

Via online access (http://cancer.digitalslidearchive.net) and mScope Portal (Aurora Interactive, Montréal, Canada) three investigators with expertise in urogenital pathology (C.K., Th.v.d.K., and G.v.L.) reviewed available whole-slide images of frozen sections of both TCGA (*n* = 260) and CPC-GENE (*n* = 199) cohorts. Both cohorts contained radical prostatectomy specimens without prior hormonal or radiation therapy. Each slide was reviewed for GS, tumour percentage and percentage CR/IDC. Percentage CR/IDC was defined as estimated number of CR/IDC tumour cells divided by the total number of cells present in the tissue slice. Since invasive cribriform and IDC-P were morphologically indistinguishable, they were not scored individually [13].

### Somatic copy number alterations

All statistical analyses were performed in the statistical programming language R v3.2.1 and all genomic coordinates in this manuscript are based on the latest hg19 genome build. Gene-wise log_2_ ratios for revised TCGA PRAD samples (based on Affymetrix SNP 6.0 arrays) were retrieved via the TCGA-Assembler R-package [30]. To obtain discrete values, gains or deletions of genetic regions were called if a sample’s copy number exceeded the threshold of ±log_2_(1.5/2). Similarly, a gene-by-sample matrix was obtained for all revised CPC-GENE samples based on Affymetrix OncoScan arrays as described in [17]. Percent genome altered (PGA) was calculated for both the whole genome (excluding chrX and chrY) as described in [17] and separately for individual chromosome arms. For chromosome arms, separate PGAs for amplifications and deletions were obtained by dividing the number of bases affected by a deletion/amplification by the number of bases of the respective chromosome arm, taking into account only one DNA strand as PGA does not account for the strand of CNAs. For all values, a Wilcoxon-Mann-Whitney test was performed to test for significant differences between GS categories.

For identifying CR/IDC-associated events, the TCGA cohort was used as discovery set and the CPC-GENE cohort was used for validation. We initially used all CR/IDC positive samples for our analyses, but subsequently limited the CR/IDC group to cases with at least 30% to account for possible signal losses due to dilution effects caused by non-CR/IDC tissue without CNAs. This dilution effect can be envisioned assuming that CNAs of interest are CR/IDC-associated and corresponding signals therefore mainly originate from the CR/IDC compartment of the tumour. Surrounding non-CR/IDC tissue hence does not harbor these CNAs and only contributes to background signal leading to a reduced signal-to-noise ratio when trying to detect the CNAs in a mixture of both tissues. Prior to analysis, duplicated gene names, known read-throughs, genes on non-random/haplotype chromosomes, as well as genes in pseudoautosomal regions and with missing data were removed. After these filtering steps, 22,350 and 22,420 genes remained for analysis of the TCGA and CPC-GENE cohort, respectively. Next, adjacent genes exhibiting the same CNA profiles were grouped into regions to further reduce the number of tests. Boschloo’s exact test (one-sided, R-package ‘Exact’) was applied to regions with CNAs in at least 10% of all samples to identify events that occurred significantly more often in samples with CR/IDC. Multiple testing correction was performed via false discovery rate (FDR) and regions with a q-value below 0.05 were considered significant. To integrate both cohorts, all genes in regions that were identified as significant in the TCGA cohort were tested in the CPC-GENE cohort. Genes with a q-value below 0.1 were considered validated. A logistic regression was used to assess which individual deletion or amplification events were predictive for CR/IDC status while accounting for PGA and GS as confounding factors. To account for correlations between PGA and individual CNAs, PGA was re-calculated for each event by excluding the chromosome the particular event was located on. Visualization of results was done with BoutrosLab.plotting.general R-package (v5.6.10; P’ng et al. in review).

### ERG expression, chromothripsis and kataegis

To quantify *ERG* expression in the TCGA cohort, RSEM ‘scaled estimates’ were obtained via TCGA-Assembler and multiplied by 10^6^ to convert them to transcripts per million (TPM). Subsequently a log_10_ transformation was applied and UCSC transcript uc002yxa.2 was used to estimate *ERG* expression. Deletion events located between *TMPRSS2* and *ERG* were determined by combining deletions of the genes *ETS2*, *BACE2*, *BRWD1*, *PSMG1* and *HMGN1*. For the CPC-GENE cohort, scores for chromothripsis and kataegic regions were computed using the ShatterProof [31] and SeqKat (Fraser et al. Nature, in press) algorithms. The maximum values for each sample were used for comparison (Wilcoxon-Mann-Whitney test) to ascertain that despite their rare occurrence, any presence of these phenomena in the CPC-GENE samples could be detected and tested for association with CR/IDC.

### Somatic mutations

Automated and curated somatic mutation calls for exome sequencing data from TCGA PRAD samples were obtained via the TCGA Data Portal (https://tcga-data.nci.nih.gov/). Functional events were summarized patient-wise for each gene (i.e. multiple mutations in one gene were only counted once per patient, excluding categories ‘Silent’ and ‘RNA’). In addition, non-recurrent events and events that occurred in less than 5% of all tested samples were excluded from further analysis; all remaining gene mutations were tested for significant enrichment in CR/IDC positive samples using Boschloo’s exact test (one-sided, R-package ‘Exact’). CPC-GENE whole genome sequencing-derived SNVs (Fraser et al. Nature, in press) were filtered to only include functional mutations located in exonic regions and then processed as described above.

## Results

### Patient characteristics

**Fig. 1:**
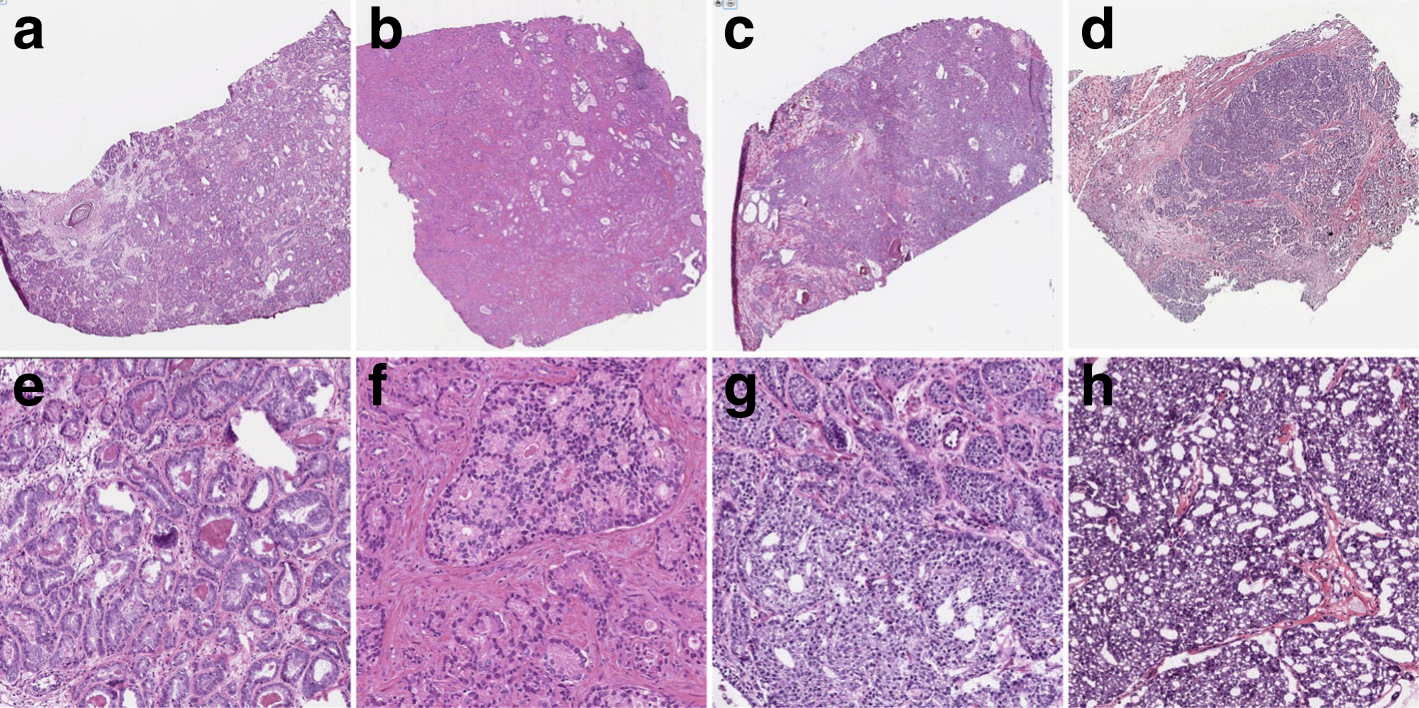
Representative images of reference HE slides of GS 6 (**a**, **e**) without CR/IDC, and GS 3 + 4 = 7 (**b**, **f**), 4 + 3 = 7 (**c**, **g**) and 4 + 4 = 8 (**d**, **h**) with CR/IDC growth

Patient characteristics of both TCGA (*n* = 260) and CPC-GENE (*n* = 199) cohorts are listed in Table 1. The TCGA cohort included more patients with adverse characteristics than the CPC-GENE cohort, having higher PSA levels (Wilcoxon rank sum test, *p* = 2.2·10^−16^), GS (Pearson’s χ2 test, *p* = 4.0·10^−5^) and pT stage (Pearson’s χ2 test, *p* = 3.1·10^−9^), which can be explained by the specific inclusion of clinically intermediate-risk disease in the latter cohort. Moreover, tumour cellularity was higher in TCGA than CPC-GENE (Additional file 1: Figure S1). Representative prostate cancer samples of GS 6 and GS ≥ 7 are depicted in Fig. 1.

**Table 1:** Clinical and pathological patient characteristics of the TCGA and CPC-GENE cohorts

	Entire cohort		CR/IDC positive		CR/IDC negative	
	TCGA	CPC-GENE	TCGA	CPC-GENE	TCGA	CPC-GENE
	Mean (IQR) or N (%)		Mean (IQR) or N (%)		Mean (IQR) or N (%)	
Number	260 (100%)	199 (100%)	80 (31%)	76 (38%)	180 (69%)	123 (62%)
Age (years)	60 (56–66)	61 (57–66)	61 (57–66)	61 (58–66)	60 (55–70)	61 (57–64)
PSA (ng/mL)	10 (5.1–11)	7.6 (4.8–9.3)	12 (6.4–15)	8.1 (4.9–10)	9.5 (4.6–9.7)	7.3 (4.8–9.1)
GS						
3 + 3	96 (37%)	69 (35%)	0	0	96 (53%)	69 (56%)
3 + 4	78 (30%)	95 (48%)	27 (34%)	44 (58%)	51 (28%)	51 (41%)
4 + 3	39 (15%)	25 (12%)	22 (27%)	22 (29%)	17 (10%)	3 (3%)
8	19 (7.3%)	9 (4%)	17 (21%)	9 (12%)	2 (1%)	0
9–10	28 (11%)	1 (1%)	14 (18%)	1 (1%)	14 (8%)	0
pT stage						
T2	112 (43%)	84 (42%)	20 (25%)	20 (26%)	92 (51%)	64 (52%)
T3a	80 (31%)	58 (29%)	28 (35%)	26 (35%)	52 (29%)	32 (26%)
T3b	55 (21%)	15 (8%)	31 (39%)	10 (13%)	24 (13%)	5 (4%)
T4	4 (2%)	0	1 (1%)	0	3 (2%)	0
Tx	9 (3%)	42 (21%)	0	20 (26%)	9 (5%)	22 (18%)

*GS* Gleason score, *PSA* Prostate Specific Antigen

### CR/IDC is associated with genomic instability

**Fig. 2:**
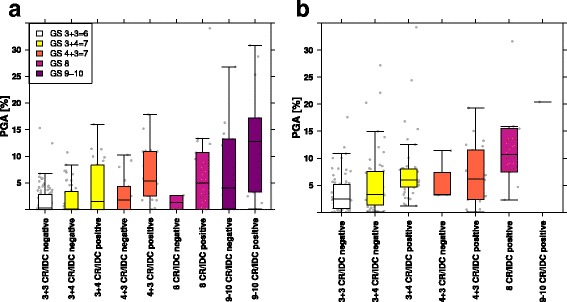
Boxplot of patient-wise PGA stratified by CR/IDC percentage and Gleason score in the TCGA (**a**) and CPC-GENE (**b**) cohort

To assess whether CR/IDC was associated with genomic instability, we calculated PGA for all patients and used a Wilcoxon-test to identify significant differences [17, 26]. PGA was 3 fold (*p* = 1.6·10^−4^) higher in men with CR/IDC as compared to men without (Fig. 2). Exclusion of men with GS 6, who generally lack CR/IDC growth, yielded similar results with 2.2 fold (p = 3·10^−4^) PGA increase in cases containing CR/IDC. Subgroup analysis revealed that PGA was significantly higher in samples with CR/IDC in GS 4 + 3 = 7 (2.2 fold; *p* = 5.3·10^−3^), but not in GS 3 + 4 = 7 (2.1 fold; *p* = 0.19), GS 8 (5.1 fold; *p* = 0.57) and GS 9–10 (1.7 fold; *p* = 0.10). Moreover, PGA scores did not differ significantly between GS 3 + 4 = 7 without CR/IDC pattern and GS 6 (1.2 fold; *p* = 0.51). Validation within the CPC-GENE cohort revealed overall 1.7 fold higher PGA of CR/IDC positive men with GS ≥ 3 + 4 = 7 (*p* = 4·10^−3^). Subgroup analysis showed 1.3 fold (*p* = 0.02) higher PGA in GS 3 + 4 = 7 cases with CR/IDC as compared to those without. PGA scores were significantly lower in GS 6 as compared to GS 3 + 4 = 7 with CR/IDC (2.2 fold; *p* = 4.7·10^−7^) than those without CR/IDC (1.6 fold; *p* = 0.07). Since 32 out of 35 CPC-GENE patients with GS ≥ 4 + 3 = 7 had CR/IDC, statistical analysis in respective subgroups lacked statistical power.

To determine whether genomic instability in CR/IDC was a global phenomenon or affected specific genomic regions, we computed PGA for individual chromosome arms utilizing deletion and amplification events independently. We found that deletions were mostly present on chromosome arms 1p, 4p, 4q, 5q, 7q, 8p, 10p, 10q, 12p, 13q, 16q, 17p, 18q and 21q in samples with CR/IDC (*p* < 0.05, Additional file 1: Figs. S2 and S3; Additional file 2: Table S1), while amplifications were found on chromosome 4q, 8p, 8q, 9p, 14q and 18p. Several of these chromosome arms have been linked to advanced prostate cancer [21, 32–35]. Increased PGA for chromosome 4p, 8p, 10q, 12p and 16q deletions were also present in the CPC-GENE cohort (*p* < 0.05, Additional file 1: Figs. S4 and S5; Additional file 2: Table S1).

### Somatic CNAs associated with aggressive clinical outcome are enriched in CR/IDC

**Fig. 3:**
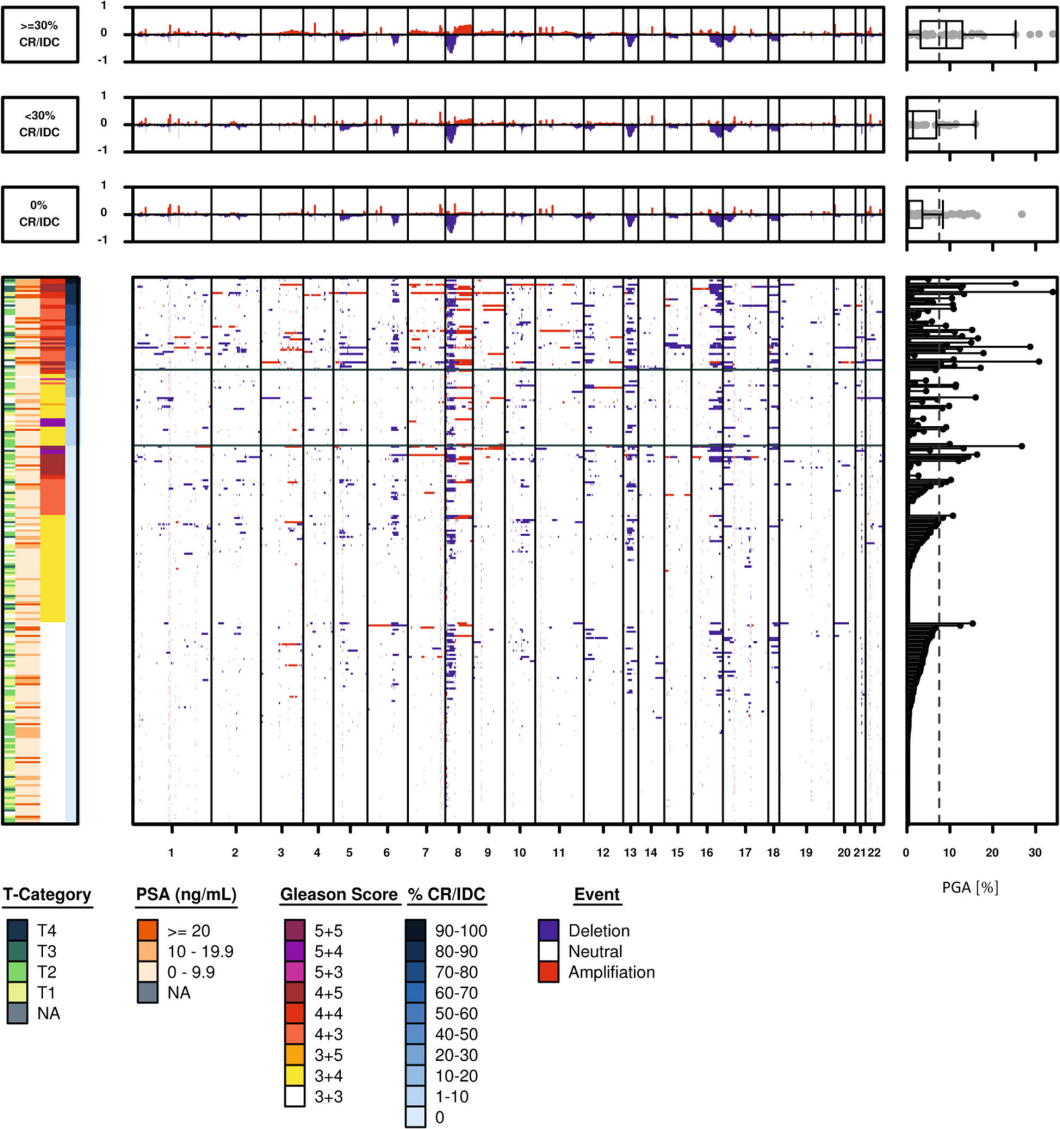
Overview heatmap of CNA in TCGA cohort. Clinical variables are displayed on the left, while PGA is displayed on the right. Samples are ordered by CR/IDC percentage, with two thresholds chosen to discriminate between negative (0%), intermediate (1–30%) and high (>30%) CR/IDC growth pattern

To identify somatic CNAs associated with CR/IDC, we applied Boschloo’s exact test, independently for each gene locus in GS ≥ 3 + 4 = 7 samples. We found 592 gene deletions and 366 amplifications significantly associated with CR/IDC (q < 0.05). These events clustered in specific chromosomal regions known to be associated with aggressive disease such as deletions of 8p (*PPP2R2A*, *NKX3–1*) [36–38], 16q22 (*CDH1*) [39], 16q23 (*BCAR1*, *CTRB1*, *CTRB2, WWOX* and *MAF*) [15, 40, 41], 16q24 [42], 10q23 (*PTEN*) [43, 44], 17p13 and 18q21 (*CCBE1*) [45] as well as amplification of 8q24 (*MYC* and *LY6* family members [15, 46, 47], Fig. 3 and Additional file 3: Table S2).

Since it was unclear whether genomic alterations occurred specifically in CR/IDC structures or also in non-cribriform prostate cancer glands adjacent to CR/IDC, we excluded samples with <30% CR/IDC growth pattern. Comparing GS ≥ 3 + 4 = 7 men with ≥30% CR/IDC (*n* = 44) to those without (*n* = 84) resulted in a total of 1299 significant deletions and 369 amplifications. Additional deletions in cases with ≥30% CR/IDC included the “Down syndrome critical region” located between *ERG* and *TMPRSS2* on 21q22 [48], 16q22 (*CTCF*) [49], 13q14 (*RB1*) [50, 51], 17p13 (*TP53*) [52], and parts of 6q [53, 54] (Additional file 4: Table S3). Although genetic deletions of genes located between the *TMPRSS2* promoter and *ERG* occurred more frequently in CR/IDC cases, we were unable to find a significant difference in *ERG* mRNA expression (Additional file 1: Figure S6). This paradoxical finding might be explained by relatively more frequent genomic translocation than deletion mechanism for *TMPRSS2:ERG* corresponding to lower genomic instability in cases without CR/IDC [55].

A trend towards lower q-values was observed when excluding tumours with <30% CR/IDC pattern suggesting that signal strength from CR/IDC specific events was diluted in cases with low CR/IDC quantity. Subsequent analyses were all performed using CR/IDC samples with at least 30% cribriform architecture. In total 474 deleted and 328 amplified genes were validated in the CPC-GENE cohort (q < 0.1), located on chromosomes 8p, 10q23, 13q22, 16q23–24, 17p13, 21q22, as well as 8q24, respectively (Additional file 5: Table S4 and Additional file 6: Figure S7). We noticed that q-values were generally lower in TCGA as compared to CPC-GENE, regardless of whether a threshold on CR/IDC was applied or not, indicating relatively lower statistical power of the latter cohort.

Since genomic instability and GS might act as confounding factors in assessing CNA events, we performed logistic regression analysis correcting for GS and PGA based on the 1668 previously identified events. A total of 779 gene deletions and 317 amplifications were independently associated with CR/IDC (q < 0.1, Additional file 7: Table S5). Deletions were mostly located on 8p21–23, 13q14, 16q21–24 as well as 18q21–23, but also included the genomic loci containing *PTEN* (10q23) [56], *RYBP*/*FOXP1* (3p13) [16] and *CASP8AP2* (6q15) [57]. The *PPP2R2A*/*BNIP3L*/*PNMA2* locus (8p21) [36] featured the lowest q-value for deletions (*p* = 0.00018, q = 0.02, OR = 10.2, 3.24–38), while the *MAFA*/*PTP4A3* locus on 8q24 did for amplifications (*p* = 0.007, q = 0.08, OR = 7.77, 1.98–41.95) [58, 59]. For CPC-GENE, logistic regression did not yield significant results after correcting for multiple comparisons, which can be attributed to lower statistical power and significant differences in pathological features.

### Somatic SNVs are not main driver events for CR/IDC growth

To identify genes affected by functional SNVs we used TCGA exome sequencing data (https://tcga-data.nci.nih.gov/) of samples with GS ≥ 7, and compared 88 samples with ≥30% CR/IDC against 143 without. Filtering for genes that harboured SNVs in at least 5% of all samples, *FOXA1* (15% versus 5%; p = 0.007), *TP53* and *SPOP* (both 19% versus 10%; *p* = 0.035) showed significantly higher mutation rates in cases with CR/IDC compared to those without (Boschloo’s exact test). Although SNV data were available for CPC-GENE samples, the number of cases, i.e. 8 with and 30 without CR/IDC was too low for statistical analysis. We did not find significant differences in overall frequency or total number of affected genes with functional SNVs (*data not shown*), indicating that SNVs are unlikely to be driver events for CR/IDC growth.

Finally, we investigated whether recently discovered DNA repair-related phenomena were linked to CR/IDC [60, 61]. We utilized available computational scores for kataegis, a pattern of localized hypermutation, and chromothripsis, a catastrophic event during which single chromosome arms or entire chromosomes are rearranged and/or lost. No statistically significant differences could be identified between cases with and without CR/IDC albeit sample numbers were low (*data not shown*).

## Discussion

Recent studies have indicated the clinical importance of both invasive cribriform and intraductal carcinoma of the prostate [6, 13, 14]. In the current study, we hypothesized that CR/IDC represents a morphologic substrate of genomic alterations associated with aggressive disease. We found that CR/IDC was associated with increased genomic instability together with chromosomal deletions of 3p13, 6q15, 8p21–23, 10q23, 13q14, 16q21–24, 18q21–23, and amplification of 8q24. The genetic losses and amplifications included several genes related to aggressive prostate cancer such as loss of *PTEN*, *RB1*, *TP53* and amplification of *MYC*. Altogether, these findings support our hypothesis that CR/IDC is a specific morphologic substrate of genomic alterations associated with aggressive disease.

Our study is in line with previous studies on genetic abnormalities related to CR/IDC growth. Dawkins et al. [62] and Bettendorf et al. [63] observed more frequently loss of heterozygosity (LOH) in IDC than in the invasive prostate cancer component. Qian et al. showed gain of chromosomes 7, 12, and Y, loss of chromosome 8, and amplification of *c-MYC* in cribriform cancer compared to other Gleason grade 3 and 4 patterns [64]. In a meta-analysis on recurrent CNAs, Williams et al. [33] compared 568 primary prostate cancer tumour samples from 8 previous studies [16, 19, 20, 65–69] with 115 metastatic prostate cancer samples from 5 studies [16, 22, 67, 70, 71]. Strikingly, the prevalence of recurrent CNAs in metastatic prostate cancers corresponded with several of the CNAs found enriched in CR/IDC, such as *PTEN* and *NKX3–1*. Recently, Chua et al. studied differences in RNA expression in prostate cancer with and without CR/IDC. They found that the long non-coding RNA SChLAP1, which has been associated with tumour progression, was significantly higher in CR/IDC, and that CR/IDC growth was associated with hypoxia [72–74]. Together these findings further support a strong relation of CR/IDC with molecular tumour progression. On the other hand, we did not find a statistically significant difference between GS 3 + 4 = 7 without CR/IDC and GS 6 cases, which further supports the question whether it is clinically relevant to distinguish CR/IDC-negative GS 3 + 4 = 7 from GS 6 prostate cancer cases.

Although prostate cancer with CR/IDC showed increased genomic instability, it is not yet clear to what extent these molecular alterations are exclusively present in CR/IDC tumour glands or whether these alterations can also be found in surrounding non-cribriform tumour glands. Using RNA in situ hybridization, we previously found that SChLAP1 was not only over-expressed in CR/IDC structures but also in adjacent non-cribriform cancer glands suggesting that it represents a field effect during tumour progression and not a specific characteristic of CR/IDC growth [72, 75]. In our study, CR/IDC was more frequently present in cases with higher GS. To exclude that genomic alterations were merely relating to higher GS and not to CR/IDC per se, we performed PGA subgroup analysis and logistic regression for CNAs, which indeed revealed an independent associated with CR/IDC in the TCGA cohort. Further comparisons of microdissected growth patterns within individual patients are mandatory to determine what events are specific for CR/IDC and which represent general effects of progression.

Elucidation of the molecular alterations associated to CR/IDC is not only of interest for molecular-biology, but might also have future impact for prostate cancer diagnosis and management. Prostate biopsies only sample a limited volume of the entire tumour and might be false-negative for CR/IDC due to sampling artefact. Since IDC represents an extensive proliferation of neoplastic cells within pre-existent acini which connect with the urethra, we postulate that these cells and/or their DNA can be shed into urine. Identification of molecular alterations associated with CR/IDC in voided urine could form the base of non-invasive tests for detection of aggressive CR/IDC.

The current study has several limitations. While we set out to validate our findings in an independent cohort, we noticed that many events originally found in the TCGA cohort could not be confirmed in the CPC-GENE dataset. This may be explained by differences in cohort composition, since the TCGA was enriched for tumours with adverse pathologic features. In addition, the statistical power of the CPC-GENE cohort was lower than of the TCGA, as its study population was smaller, included samples with lower and more variable tumour percentage, and was strongly enriched for CR/IDC in GS 8–10. Nevertheless, both datasets independently revealed the association of CR/IDC with increased genomic instability and the deletions of various specific genomic regions and genes. Furthermore, tumour heterogeneity and sampling artefacts may have also influenced the outcome of this study, as our current data was based on DNA derived from a freshly frozen section per patient. Hence, there may have been, for instance, CR/IDC growth in an adjacent region that was not sampled for genomic analysis that may have been detected due to a field effect. This might be the cause of the relatively small effect sizes in the current study. Lastly, we did not independently analyse CR/IDC growth in relation to adjacent tumour glands using, for instance, laser-capture microdissection or in situ hybridization.

## Conclusion

We found that pathologic CR/IDC growth pattern is associated genomic instability including deletions of 8p, 10q23, 13q22, 16q22–24, 17p13 and 21q22, as well as smaller 8q24 amplification. These results indicate that CR/IDC is a histopathological substrate of molecular tumour progression and present a rationale for its aggressive clinical behaviour.

## Additional files

Additional file 1: **Figure S1.** Comparison of tumour cell percentage in whole-slide reference images for both TCGA and CPC-GENE cohorts, stratified by CR/IDC status. **Figure S2.** PGA for deletion events in the TCGA cohort per chromosome arm for GS ≥ 3 + 4 = 7 with and without CR/IDC. **Figure S3.** PGA for amplification events in the TCGA cohort per chromosome arm for GS ≥ 3 + 4 = 7 with and without CR/IDC. **Figure S4.** PGA for deletion events in the CPC-GENE cohort per chromosome arm for GS ≥ 3 + 4 = 7 with and without CR/IDC. **Figure S5.** PGA for amplification events in the CPC-GENE cohort per chromosome arm for GS ≥ 3 + 4 = 7 with and without CR/IDC. **Figure S6.** Overview of *ERG* expression in TCGA [log_10_(TPM)] stratified by CR/IDC status (A) and deletion of the genomic region between *TMPRSS2* and *ERG* (B). (PDF 3140 kb)

Additional file 2: Table S1. Overview of genomic instability of individual chromosome arms in both TCGA and CPC-GENE datasets. Genomic instability was calculated based on a modified PGA formula (see methods). *P*-values are based on a Wilcon–Mann–Whitney test while log2FC represents the log_2_ ratio of the average PGA scores for CR/IDC positive samples and CR/IDC negative samples. PGA scores for deletions and amplifications were calculated and tested separately. (XLS 139 kb)

Additional file 3: Table S2. Gene-wise copy number alterations associated with CR/IDC growth using any CR/IDC presence for patient stratification. Columns contain: *Symbol* – official gene symbol, *Chromosome* / *Start* / *End* – genomic coordinates of gene locus, *FDR* – Boschloo’s exact test *p*-value after correcting for multiple tests using the Benjamini–Hochberg procedure. *amplifications_case* – number of CR/IDC positive samples with an amplification spanning gene locus, *amplifications_control* – number of control samples with an amplification spanning gene locus, *cases* – total number of CR/IDC positive samples, *controls* – total number of control samples. All entries are sorted by genomic location. Deletions are presented in the same format and listed separately. (XLS 226 kb)

Additional file 4: Table S3. Gene-wise copy number alterations associated with CR/IDC growth using a ≥ 30% CR/IDC threshold to stratify samples. Columns contain: *Symbol* – official gene symbol, *Chromosome* / *Start* / *End* – genomic coordinates of gene locus, *FDR* – Boschloo’s exact test *p*-value after correcting for multiple tests using the Benjamini–Hochberg procedure. *amplifications_case* – number of CR/IDC positive samples with an amplification spanning gene locus, *amplifications_control* – number of control samples with an amplification spanning gene locus, *cases* – total number of CR/IDC positive samples, *controls* – total number of control samples. All entries are sorted by genomic location. Deletions are presented in the same format and listed separately. (XLS 161 kb)

Additional file 5: Table S4. Gene-wise copy number alterations detected in the TCGA cohort and validated in the CPC-GENE cohort using a ≥ 30% CR/IDC threshold to stratify samples. Columns contain: *Symbol* – official gene symbol, *Chromosome* / *Start* / *End* – genomic coordinates of gene locus, *FDR* – Boschloo’s exact test p-value after correcting for multiple tests using the Benjamini–Hochberg procedure for specified dataset. *amplifications_case* – number of CR/IDC positive samples in specified dataset with an amplification spanning gene locus, *amplifications_control* – number of control samples in specified dataset with an amplification spanning gene locus, *cases* – total number of CR/IDC positive samples in specified dataset, *controls* – total number of control samples in specified dataset. All entries are sorted by genomic location. Deletions are presented in the same format and listed separately. (PDF 12328 kb)

Additional file 6: Figure S7. Overview heatmap of copy number alterations in CPC-GENE cohort. Clinical variables are displayed on the left, while percent genome altered (PGA) is displayed on the right. Samples are ordered by CR/IDC percentage, with two thresholds chosen to discriminate between negative (0%) and intermediate (< 30%) CR/IDC status. (XLSX 14 kb)

Additional file 7: Table S5. Significant CNAs identified by logistic regression analysis accounting for genomic instability as confounding factor in the TCGA dataset. Columns contain: *Symbol* – official gene symbol, *Chromosome* / *Start* / *End* – genomic coordinates of gene locus, *p-alue* / *FDR* – p-value of logistic regression before and after correction for multiple tests via FDR, *odds ratio* / 95% CI – odds ratio and 95% confidence interval based on logistic regression. Deletions and amplifications are presented in the same format and listed separately. All entries are sorted by genomic location. (XLS 266 kb)
