# Supplementary material for: Cribriform and intraductal prostate cancer are associated with increased genomic instability and distinct genomic alterations
Source: BMC Cancer. 2018 Jan 2;18:8. doi: 10.1186/s12885-017-3976-z (PMC5751811; doi:10.1186/s12885-017-3976-z)

Supplementary Figure 1

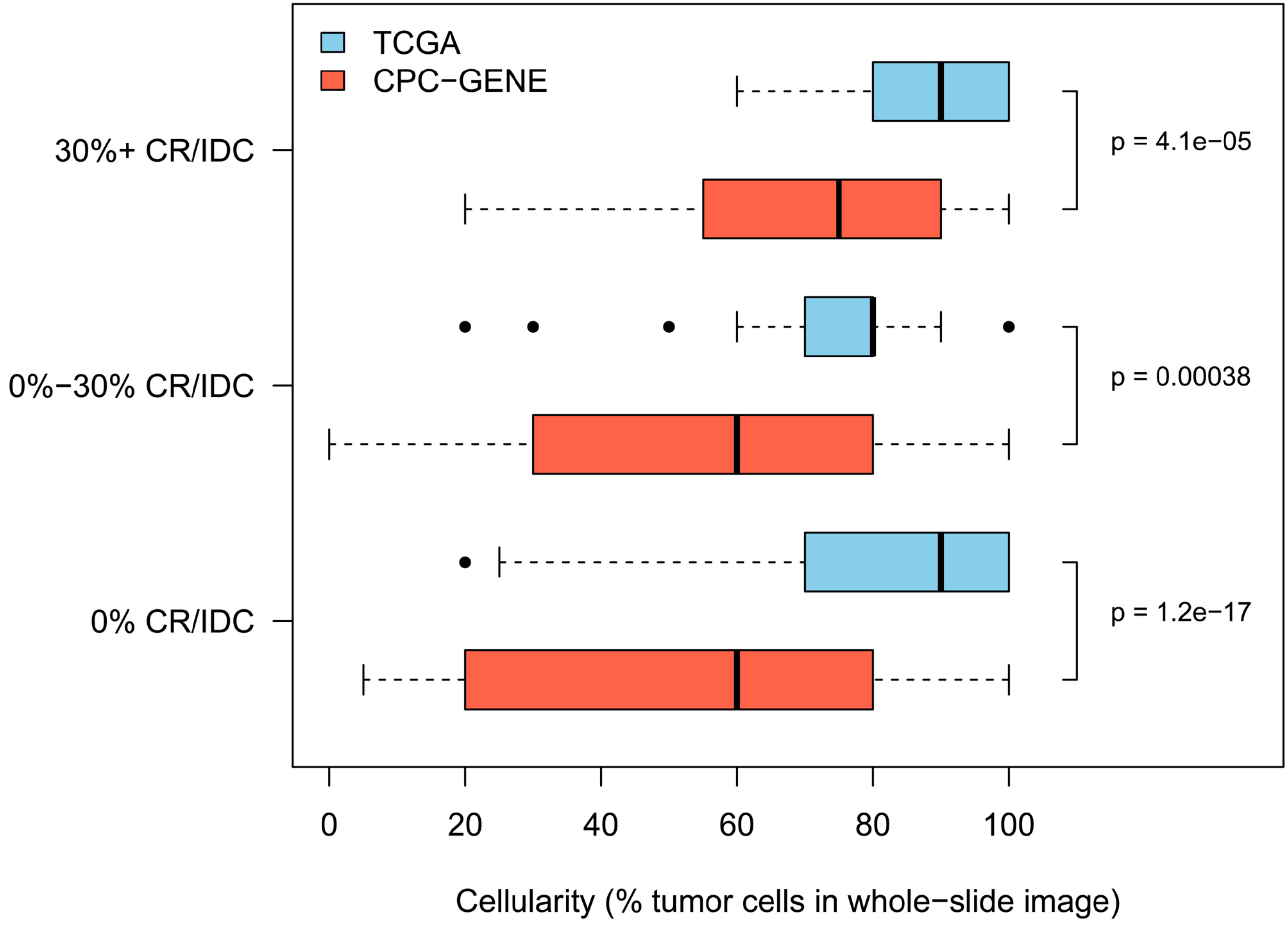

Supplementary Figure 2

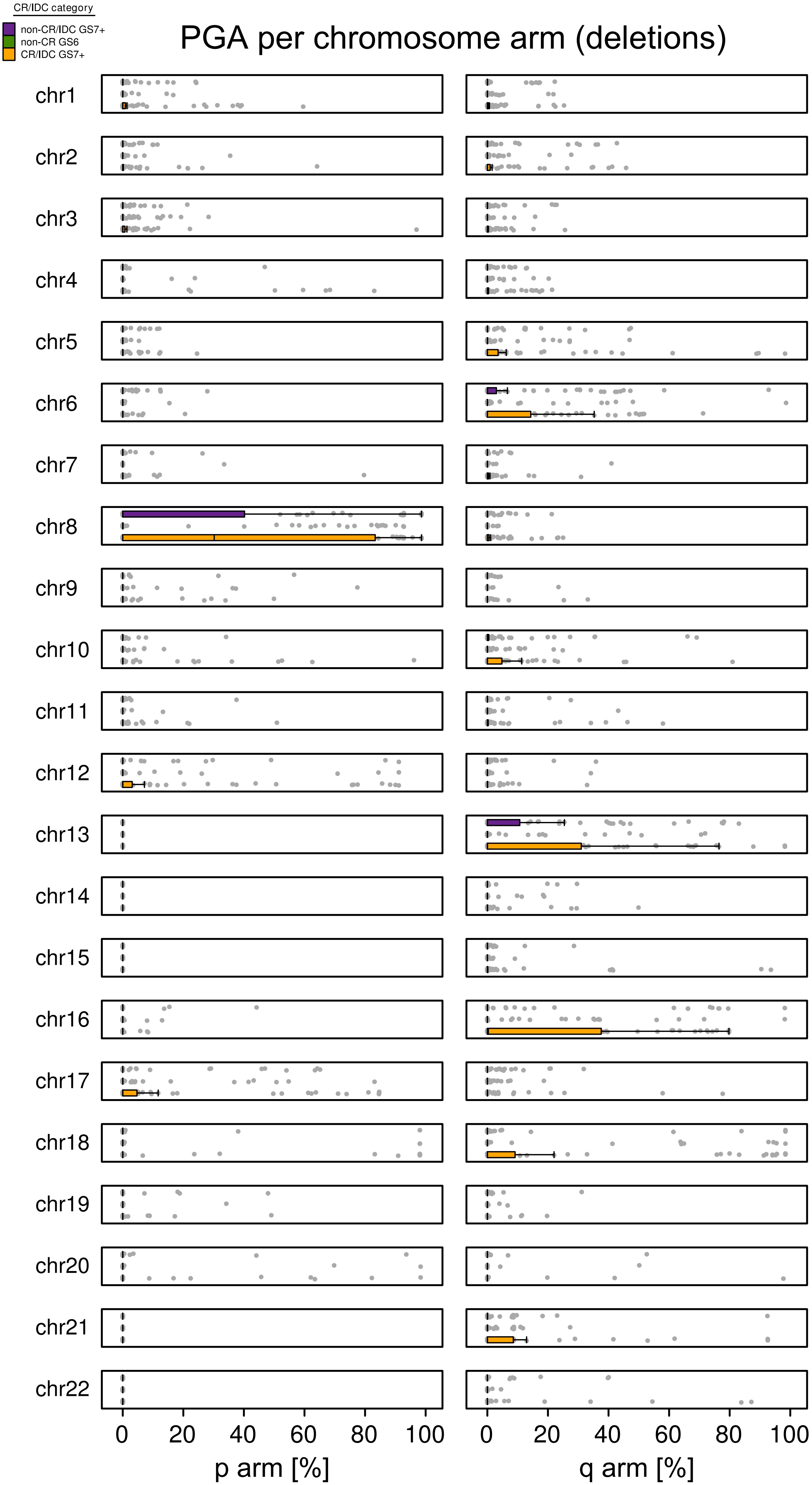

Supplementary Figure 3

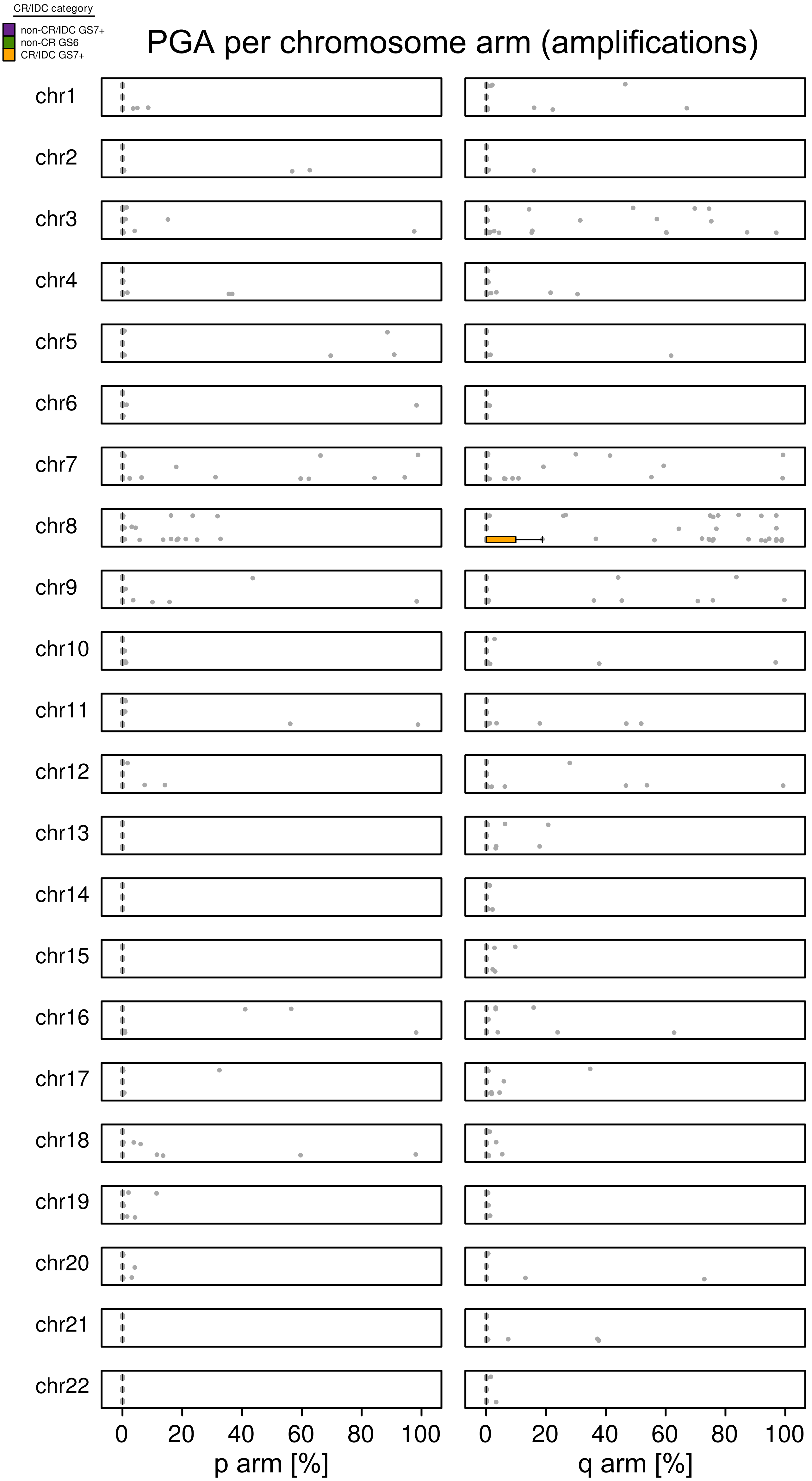

Supplementary Figure 4

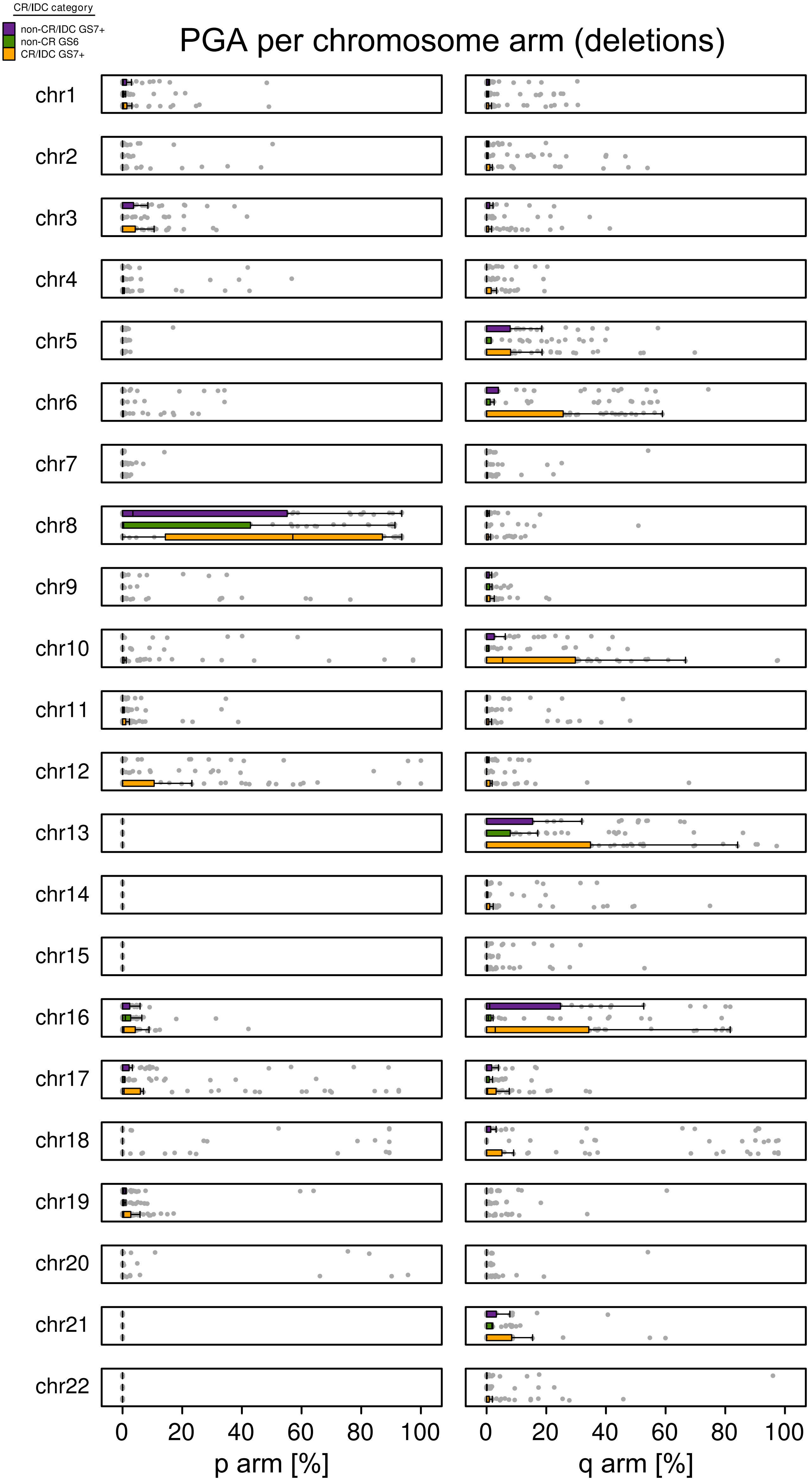

Supplementary Figure 5

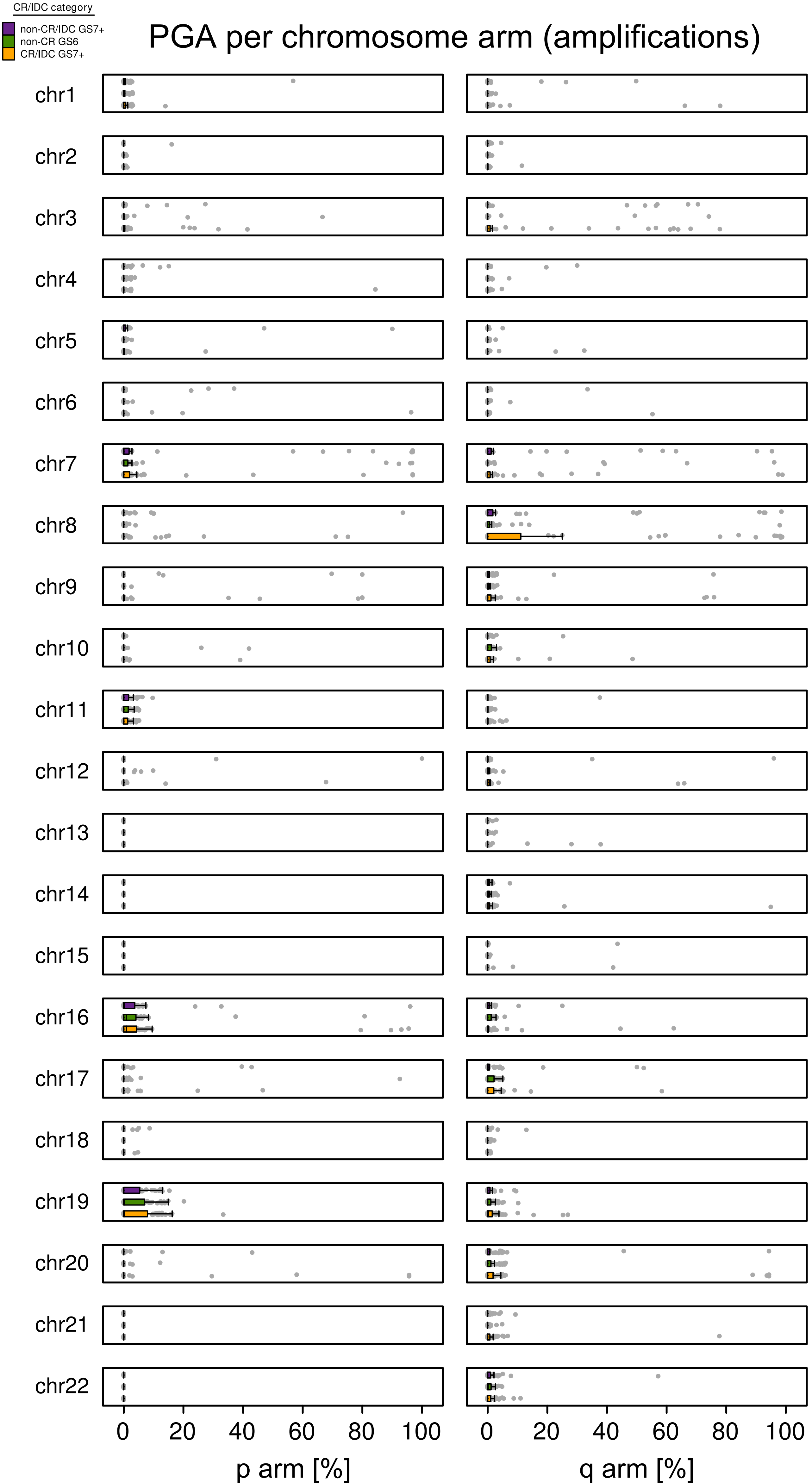

Supplementary Figure 6

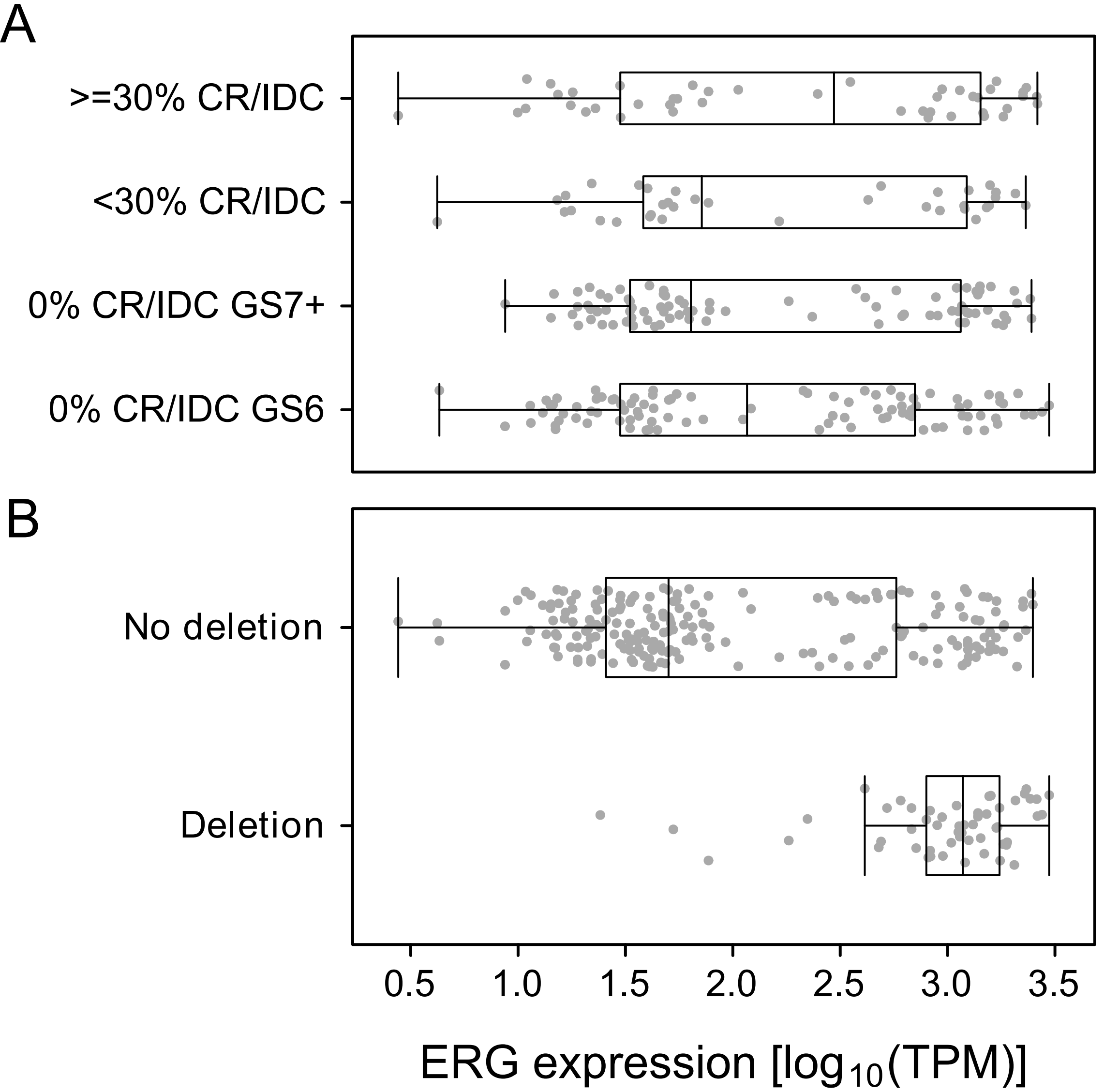

Supplement: Supplementary file 1 — Figure S1. Comparison of tumour cell percentage in whole-slide reference images for both TCGA and CPC-GENE cohorts, stratified by CR/IDC status. Figure S2. PGA for deletion events in the TCGA cohort per chromosome arm for GS ≥ 3 + 4 = 7 with and without CR/IDC. Figure S3. PGA for amplification events in the TCGA cohort per chromosome arm for GS ≥ 3 + 4 = 7 with and without CR/IDC. Figure S4. PGA for deletion events in the CPC-GENE cohort per chromosome arm for GS ≥ 3 + 4 = 7 with and without CR/IDC. Figure S5. PGA for amplification events in the CPC-GENE cohort per chromosome arm for GS ≥ 3 + 4 = 7 with and without CR/IDC. Figure S6. Overview of ERG expression in TCGA [log10(TPM)] stratified by CR/IDC status (A) and deletion of the genomic region between TMPRSS2 and ERG (B). (PDF 3140 kb) [file 12885_2017_3976_MOESM1_ESM.pdf]
