# Supplementary figures and images for: Cribriform and intraductal prostate cancer are associated with increased genomic instability and distinct genomic alterations
Source: BMC Cancer. 2018 Jan 2;18:8. doi: 10.1186/s12885-017-3976-z (PMC5751811; doi:10.1186/s12885-017-3976-z)

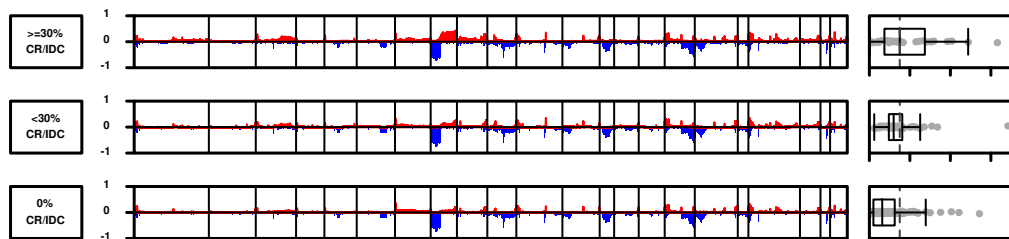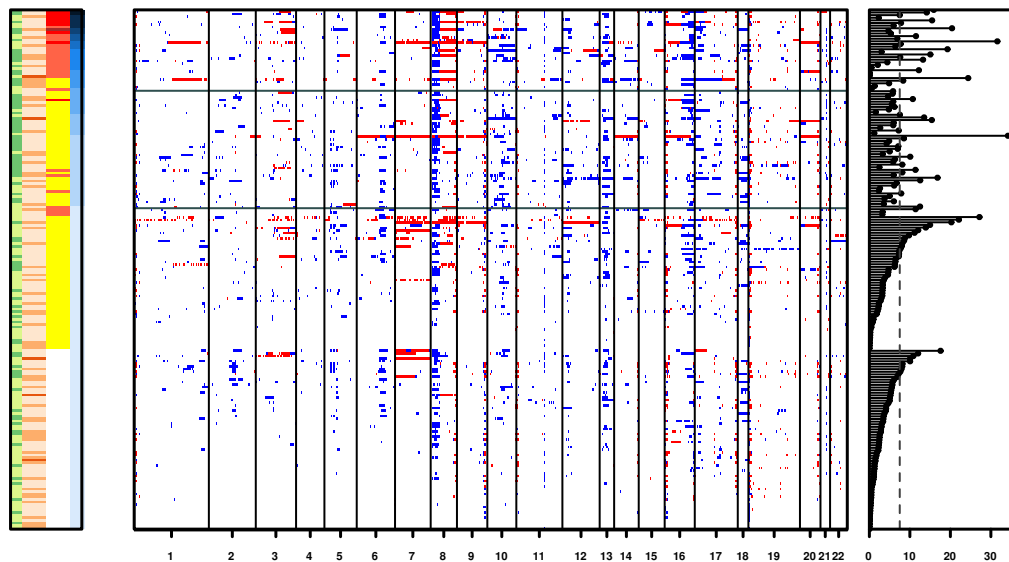

T-Category

T2  
T1  
NA

PSA (ng/mL)

$\geq 20$   
10 - 19.9  
0 - 9.9  
NA

Gleason Score

4+5  
4+4  
4+3  
3+4  
3+3

% CR/IDC

90-100  
80-90  
70-80  
60-70  
50-60  
40-50  
30-40  
20-30  
10-20  
1-10  
0

Event

Deletion  
Neutral  
Amplification

PGa [%]

Supplement: Supplementary file 6 — Overview heatmap of copy number alterations in CPC-GENE cohort. Clinical variables are displayed on the left, while percent genome altered (PGA) is displayed on the right. Samples are ordered by CR/IDC percentage, with two thresholds chosen to discriminate between negative (0%) and intermediate (< 30%) CR/IDC status. (XLSX 14 kb) [file 12885_2017_3976_MOESM6_ESM.pdf]
